# A Network Pharmacology and Molecular Docking Strategy to Explore Potential Targets and Mechanisms Underlying the Effect of Curcumin on Osteonecrosis of the Femoral Head in Systemic Lupus Erythematosus

**DOI:** 10.1155/2021/5538643

**Published:** 2021-09-13

**Authors:** Pan Kang, Zhiming Wu, Yue Zhong, Zihao Wang, Chi Zhou, Shaochuan Huo, Hai Guo, Songtao Li, Kun Xu, Lingyun Liu, Shuai Chen, Hongyu Tang, Haibin Wang

**Affiliations:** ^1^Guangzhou University of Chinese Medicine, Guangzhou 510405, China; ^2^Shenzhen Hospital (Futian) of Guangzhou University of Chinese Medicine, No. 6001, North Ring Road, Futian District, Shenzhen City, Guangdong Province, 518048, China; ^3^Queen's University Belfast, BT71NN, UK; ^4^Department of Orthopaedic Surgery, First Affiliated Hospital, Guangzhou University of Chinese Medicine, Guangzhou 510405, China; ^5^Liuzhou Traditional Chinese Medicine Hospital (Liuzhou Zhuang Medical Hospital), No. 6, Red Gourd, Donghuan Avenue, Chengzhong District, Liuzhou City, Guangxi Province 545000, China; ^6^Shi's Center of Orthopedics and Traumatology, Shuguang Hospital Affiliated to Shanghai University of Traditional Chinese Medicine, Institute of Traumatology & Orthopedics, Shanghai Academy of Traditional Chinese Medicine, China; ^7^College of Basic Medicine, Guangzhou University of Chinese Medicine, Guangzhou 510006, China

## Abstract

**Background:**

Systemic lupus erythematosus (SLE) is a refractory immune disease, which is often complicated with osteonecrosis of the femoral head (ONFH). Curcumin, the most active ingredient of Curcuma longa with a variety of biological activities, has wide effects on the body system. The study is aimed at exploring the potential therapeutic targets underlying the effect of curcumin on SLE-ONFH by utilizing a network pharmacology approach and molecular docking strategy.

**Methods:**

Curcumin and its drug targets were identified using network analysis. First, the Swiss target prediction, GeneCards, and OMIM databases were mined for information relevant to the prediction of curcumin targets and SLE-ONFH-related targets. Second, the curcumin target gene, SLE-ONFH shared gene, and curcumin-SLE-ONFH target gene networks were created in Cytoscape software followed by collecting the candidate targets of each component by R software. Third, the targets and enriched pathways were examined by Gene Ontology (GO) and Kyoto Encyclopedia of Genes and Genomes (KEGG) pathway enrichment analysis. Eventually, a gene-pathway network was constructed and visualized by Cytoscape software; key potential central targets were verified and checked by molecular docking and literature review.

**Results:**

201 potential targets of curcumin and 170 related targets involved in SLE-ONFH were subjected to network analysis, and the 36 intersection targets indicated the potential targets of curcumin for the treatment of SLE-ONFH. Additionally, for getting more comprehensive and accurate candidate genes, the 36 potential targets were determined to be analyzed by network topology and 285 candidate genes were obtained finally. The top 20 biological processes, cellular components, and molecular functions were identified, when corrected by a *P* value ≤ 0.05. 20 related signaling pathways were identified by KEGG analysis, when corrected according to a Bonferroni *P* value ≤ 0.05. Molecular docking showed that the top three genes (TP53, IL6, VEGFA) have good binding force with curcumin; combined with literature review, some other genes such as TNF, CCND1, CASP3, and MMP9 were also identified.

**Conclusion:**

The present study explored the potential targets and signaling pathways of curcumin against SLE-ONFH, which could provide a better understanding of its effects in terms of regulating cell cycle, angiogenesis, immunosuppression, inflammation, and bone destruction.

## 1. Introduction

**Figure 1:**
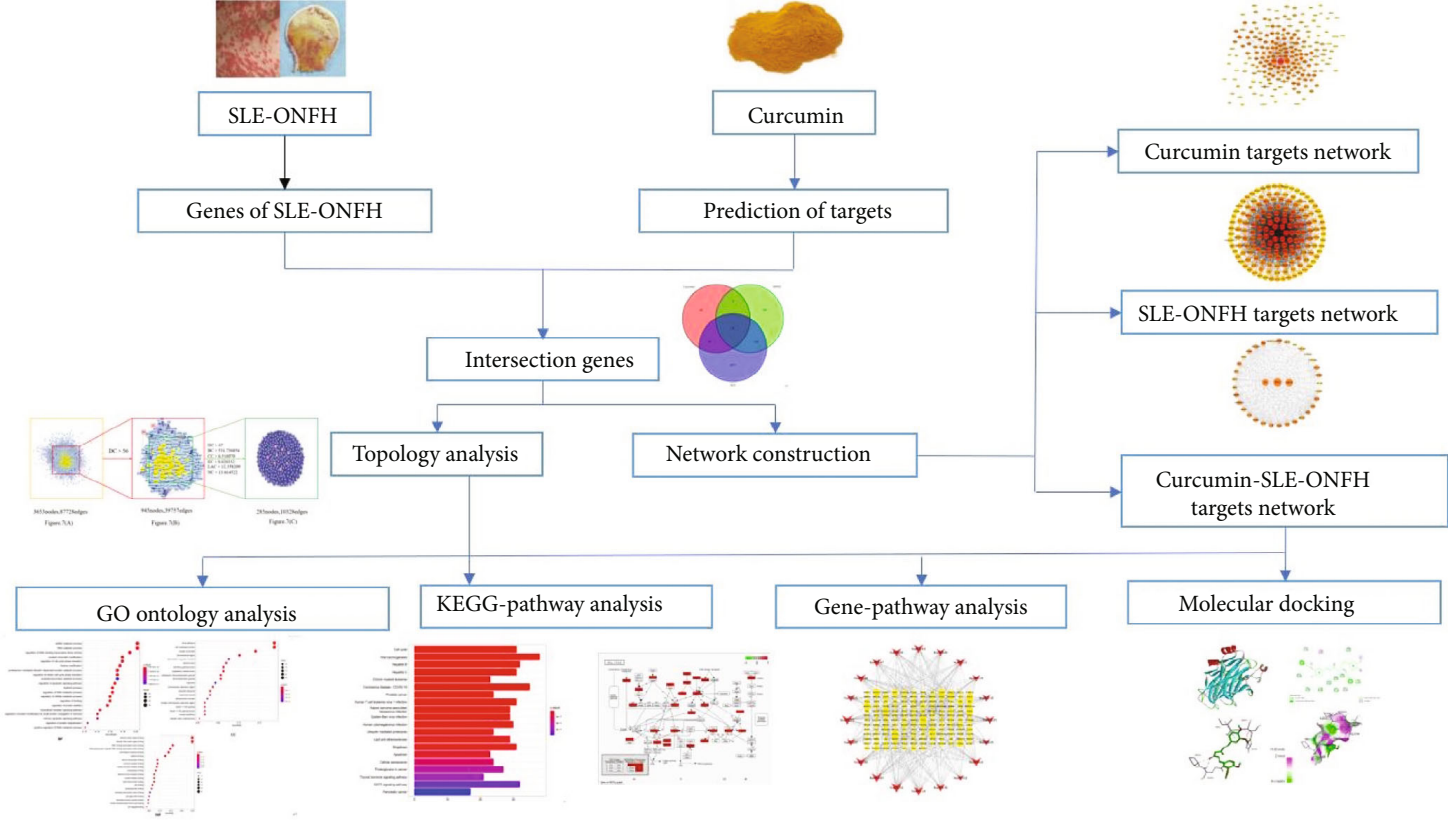
Workflow of the systematic strategies to elucidate the mechanisms of curcumin in the treatment of SLE-ONFH. ① Acquisition of drugs and disease targets. ② Collection of curcumin targets for the treatment of SLE-ONFH. ③ Analysis of therapeutic target proteins. ④ Topology of analysis of therapeutic targets. ⑤ The construction of curcumin target network, SLE-ONFH target network, and curcumin-SLE-ONFH target protein-protein interaction (PPI) network. ⑥ GO and KEGG enrichment analysis. ⑦ Gene-pathway network analysis. ⑧ Molecular docking.

Systemic lupus erythematosus (SLE) is a chronic autoimmune disease, which prevalence ranges from 20 to 150 cases per 100,000 population and appears to be increasing as the disease is recognized more readily and survival increases [1]. SLE can cause multiple organ dysfunction such as the lung, kidney, blood vessel, and joint; the diverse clinical manifestations of SLE present a challenge to the clinician [2]. Osteonecrosis (ON) remains a serious complication in SLE with prevalence ranging from 10% to 50%, and the femoral head is the most common site [3–9]. The pathogenesis of SLE-ONFH has not yet been fully elucidated. Except for joint arthroplasty in the late inactive phase, the active phase usually requires effective drugs or other measures for SLE itself, which is more complicated. To date, the drug for SLE is mainly glucocorticoids with immunosuppressive effect, which have achieved good therapeutic effects. However, some side effects have resulted because of their extensive use. A large number of studies have reported that the use of glucocorticoids can cause femoral head necrosis (ONFH) [10–12] and long-term high-dose cyclophosphamide can induce infection, reproductive toxicity, and other adverse reactions [13, 14]. Obviously, none of these seems to be an ideal treatment for SLE-ONFH in the future. Traditional Chinese medicine (TCM) has the characteristics of multitarget, multicomponent, multifunction, and generally low toxicity or side effects [15–18]. It has been widely used in the treatment of various diseases including immune diseases [19, 20] and has attracted more and more attention in recent years. The Chinese medicine arsenic being found to be effective in treating leukemia is one of the best examples [21]. Curcumin, an effective monomeric component mainly extracted from the traditional Chinese medicine turmeric, has been reported to have protective effects on SLE and ONFH [22–25]. However, its potential targets and mechanisms for the treatment of SLE-ONFH are not still very clear, and there is no systematic and comprehensive understanding of the relationship between therapeutic targets and pathways. With the improvement of bioinformatics and related databases, network pharmacology and molecular docking strategies are widely used in drug research, which revealed the interrelationship between drug-target-pathway-disease. In this study, we constructed an interaction network between SLE and ONFH, as well as their interaction network with curcumin's targets. And molecular docking technology was used to verify the potential targets of curcumin in the treatment of SLE-ONFH. In addition, relevant signal pathways are obtained by bioinformatics analysis to reveal the underlying mechanisms. The entire research workflow is shown in Figure 1.

## 2. Materials and Methods

### 2.1. Data Preparation

#### 2.1.1. Chemical Structures

**Figure 2:**
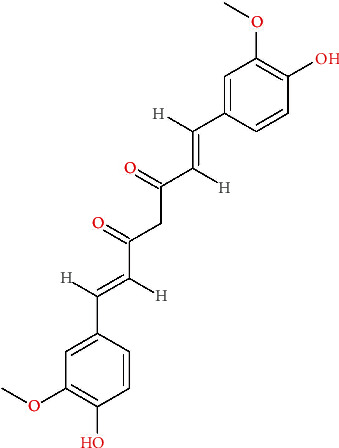
Curcumin chemical structure, CAS number: 458-37-7.

The 2D chemical structure of curcumin (Figure 2) was obtained from PubChem (http://pubchem.ncbi.nlm.nih.gov), which is an open chemical database that provides information on compound structures and descriptive data.

#### 2.1.2. Prediction of Curcumin Targets

The Swiss target prediction database (http://www.swisstargetprediction.ch/) is a web-based tool, on-line since 2014, to perform ligand-based target prediction for any bioactive small molecule [26]. To get curcumin relevant target genes, the “SMILES” format of curcumin was obtained and inputted into the Swiss target prediction database by searching curcumin in the PubChem database. In addition, the HERB database (http://herb.ac.cn/) was used to get curcumin targets for a comprehensive collection of drug targets, which is a high-throughput experiment- and reference-guided database of traditional Chinese medicine [27]. The targets are mainly from TCMID (http://www.megabionet.org/tcmid/) and TCMSP (https://tcmspw.com/tcmsp.php) databases [28, 29]. Finally, each of the predicted targets was inputted into the UniProt database (https://www.uniprot.org/) for screening. In order to increase credibility, we only need human targets which have been annotated and reviewed, and the potential targets of curcumin were identified by eliminating duplicate and nonstandard targets.

#### 2.1.3. Predict Targets of Curcumin against SLE-ONFH

The SLE and ONFH-related targets were identified in the GeneCards database (https://www.genecards.org/) using the phrase “Systemic lupus erythematosus”, “SLE”, “Osteonecrosis of Femoral Head”, and “ONFH” as the keyword. The targets were also supplemented with the OMIM database (https://omim.org/) [30]. A Venn script was installed in the R software 4.0.2 software to get targets of SLE-ONFH, which was the intersection genes of two diseases. To determine the predicted targets of curcumin relevant to SLE-ONFH, the potential targets of curcumin were taken intersection with the relevant targets involved in SLE-ONFH by R software.

### 2.2. Network Construction

#### 2.2.1. Curcumin Target Network and SLE-ONFH Target Network

The curcumin target network and SLE-ONFH network were constructed and visualized using Cytoscape 3.8.0 software [31–33]; they were uploaded to the String database (https://string-db.org/), which is including direct and indirect interactions between proteins and calculates a confidence score for all protein interactions [34, 35]. The score is proportional to the confidence in the protein interaction, ranged from 0 to 1, and we set the score greater than 0.9 to be high confidence [36, 37].

#### 2.2.2. Curcumin-SLE-ONFH Target PPI Network

Shared targets of curcumin, SLE, and ONFH were obtained by running the Venn script as described above, which was installed in the R 4.0.2 software. They were uploaded to Cytoscape software. The PPI data were obtained from the Database of Interacting Proteins (DIP™), Biological General Repository for Interaction Datasets (Bio GRID), Human Protein Reference Database (HPRD), IntAct Molecular Interaction Database (IntAct), Molecular INTeraction database (MINT), and biomolecular interaction network database (BIND) using the plugin BisoGenet of Cytoscape software [38].

### 2.3. Topology Analysis

After uploading the common gene above, the nodes with topological importance in the interaction network were screened by calculating Degree Centrality (DC), betweenness centrality (BC), closeness centrality (CC), Eigenvector Centrality (EC), local average connectivity-based method (LAC), and Network Centrality (NC) with the Cytoscape plugin CytoNCA. At the heart of Cytoscape is a network. A simple network diagram consists of nodes and edges. Each node represents a gene. The node-node connection represents the interaction between these nodes. DC believes that the greater the number of neighbors of a node, the greater its impact. CC was used to calculate the importance of nodes. BC is a kind of centrality measure. EC is an indicator in ranking the importance of nodes in the network. The closer the EC of a node is to the network radius, the closer the node is to the network center. LAC is often used to determine the importance of proteins by assessing the relationship between proteins and their neighbors. NC is a method based on the edge clustering coefficient, which considers not only the centrality of nodes but also the relationship between nodes and adjacent nodes [39–41]. These parameters represent the topological importance, and they have been reported about their definitions and computational formulas. They were all used in network pharmacology and systems pharmacology [42].

### 2.4. Enrichment Analysis

GO analysis with the biological process, cellular component, and molecular function was carried out by R software. The result is obtained by running the script installed on R software including “colorspace,” “stringi,” “ggplot2,” “BiocManager,” “clusterProfiler,” and “enrichplot.” The top 20 items were selected and visualized; a Bonferroni-corrected *P* value ≤ 0.05 was set as the default option. The Kyoto Encyclopedia of Genes and Genomes (KEGG) pathway enrichment analysis method is similar to the GO analysis above.

### 2.5. Gene-Pathway Network Analysis

The gene-pathway network was constructed based on the significantly enriched pathways with genes that regulated these pathways. The topological analysis of 20 pathways and 285 genes was carried out with BC. The squares represented target genes, and the V-shapes represented pathways in the network.

### 2.6. Molecular Docking

Molecular docking simulations were used to explore the potential interaction between the top 3 genes of the network above. The SDF format of curcumin was downloaded from the PubChem database; Chem 3D software was used to convert the SDF format into mol2 format file; then, the RESB database was used to get the PDB format structure of the top 3 target proteins. After that, solvent molecules and ligands were removed by Pymol software and saved as pdbqt format. Docking simulations were performed in Autodock1.1.2 software, and Discovery Studio 2020 was used to visually analyze the docking conformation at last [43, 44].

## 3. Results

### 3.1. Target Network Analysis

#### 3.1.1. Curcumin Target Network

**Figure 3:**
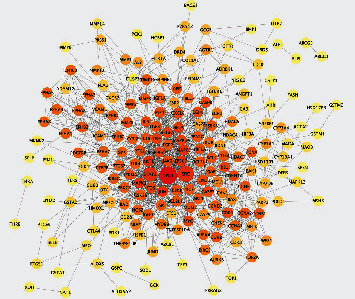
Curcumin target network. Note: PPI network of targets regulated by curcumin.

100, 152, and 11 target genes of curcumin were identified by searching the Swiss, HERB, and STITCH databases, respectively, and 240 genes were selected as curcumin target candidate genes through running R software to remove duplicate genes. In order to ensure credibility, the 240 target genes were inputted into the STRING database; by setting the highest confidence (0.9000) and selecting the condition “Homo sapiens,” 201 targets were finally identified after hiding disconnected nodes in the network. The PPI network of them was saved as “tsv” file and visualized by Cytoscape software according to the degree (Figure 3). This network represented the regulatory relationship of the curcumin targets. In the figure, the nodes represented the target points, and the edges indicated the relationships between the targets. Four important values (Table 1) average shortest path length (ASPL), betweenness centrality (BC), closeness centrality (CC), and clustering coefficient were analyzed; ASPL represented the smallest number of links that a node needed to connect the whole network. In a network, the smaller the ASPL, the more efficient the signal conduction through a node. BC represented the probability that the signal passed through the nodes, and the higher the value of BC, the more important the node. The closeness centrality (CC) and the clustering coefficient could reflect the network tightness, and a tighter network was of higher efficiency [45, 46]. The degree of a node referred to the number of edges between nodes in a network. The greater a node's degree is, the more nodes are connected to it and the more important it is in the network [47, 48]. We used the value of the degree to analyze the node's importance in the network and screening the top 30 genes, which is more than twice the median of the target genes at least [49]. It indicated that these targets might be more effective in the treatment of SLE-ONFH.

#### 3.1.2. Targets and Network of SLE-ONFH

**Figure 4:**
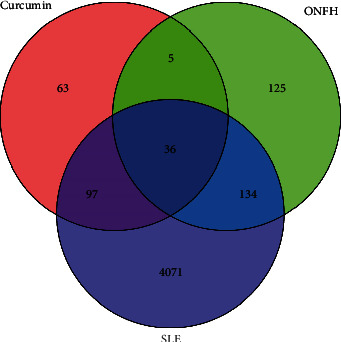
Venn diagram of curcumin, SLE, and ONFH intersection targets.

**Figure 5:**
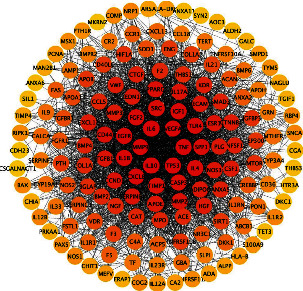
PPI network of SLE-ONFH; the closer to the center of the circle, the darker the color, and the more likely it is to be a potential target.

After removing the duplication, we obtained 4338 SLE-related targets and 300 ONFH-related targets from the GeneCards and OMIM databases, respectively. In order to improve the comprehensiveness and credibility of the data, these targets were all included in the current study. Finally, 170 intersection genes related to SLE-ONFH were obtained by running the Venn map script installed in R software, and 36 genes related to SLE, ONFH, and curcumin were also received, as shown in Figure 4. The STRING database and Cytoscape software were used to construct and visualize the PPI network of 170 genes (Figure 5); the method is similar to the description in 3.1.1.

#### 3.1.3. Curcumin-SLE-ONFH Target Network

**Figure 6:**
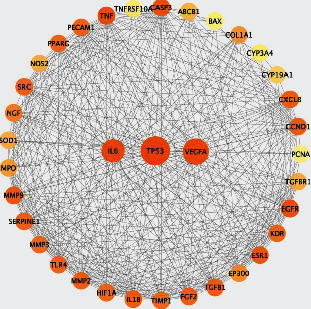
PPI network of curcumin-SLE-ONFH; the top three potential genes sorted by degree were TP53, VEGFA, and IL6.

According to the network pharmacology analysis method, 36 overlapping genes for curcumin, SLE, and ONFH were defined as potential genes (Table 2) for curcumin against SLE-ONFH. They were imported into the STRING database, and the results were saved as a “tsv” file. Then, the file was imported into Cytoscape software, and the curcumin-SLE-ONFH target network diagram was generated (Figure 6). We found that the number of nodes was 36, the number of edges was 406, the average node degree was 22.55, and the genes with a degree greater than 22.55 were represented by an ellipse in the network; they may be important, among which TP53, VEGFA, IL6, TNF, EGFR, CASP3, MMP9, etc.

### 3.2. Identification of Candidate Targets for Curcumin against SLE-ONFH Based on Topology Analysis

**Figure 7:**
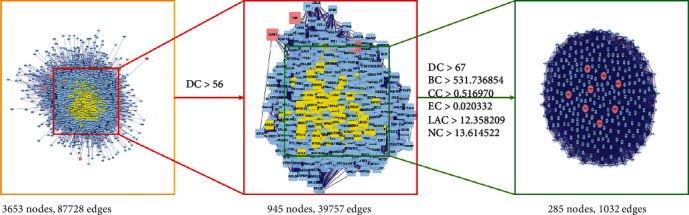
Topological analysis of 36 potential genes; 285 targets were finally identified.

To reveal the mechanisms underlying curcumin's effects on SLE-ONFH and obtain candidate genes more comprehensively and accurately, topological analysis of 36 potential genes was performed through the plugin BisoGenet and CytoNCA of Cytoscape; a network consisting of 3653 nodes and 87728 edges is presented in Figure 7(a). The median degree of all nodes was 28, and the nodes with more than 56 degrees were identified as significant targets according to the previous research [49]. A network of significant targets for curcumin against SLE-ONFH was constructed, and it contained 945 nodes and 39,757 edges (Figure 7(b)). The median values of DC, BC, CC, EC, LAC, and NC were 117, 6,403.125, 0.318309, 0.024411, 19.61376, and 27.984, respectively. The candidate targets were further screened, and 285 targets with DC > 67, BC > 531.736854, CC > 0.516970, EC > 0.020332, LAC > 12.358209, and NC > 13.614522 were identified (Figure 7(c)).

### 3.3. GO and KEGG Enrichment Analysis

#### 3.3.1. GO Analysis

**Figure 8:**
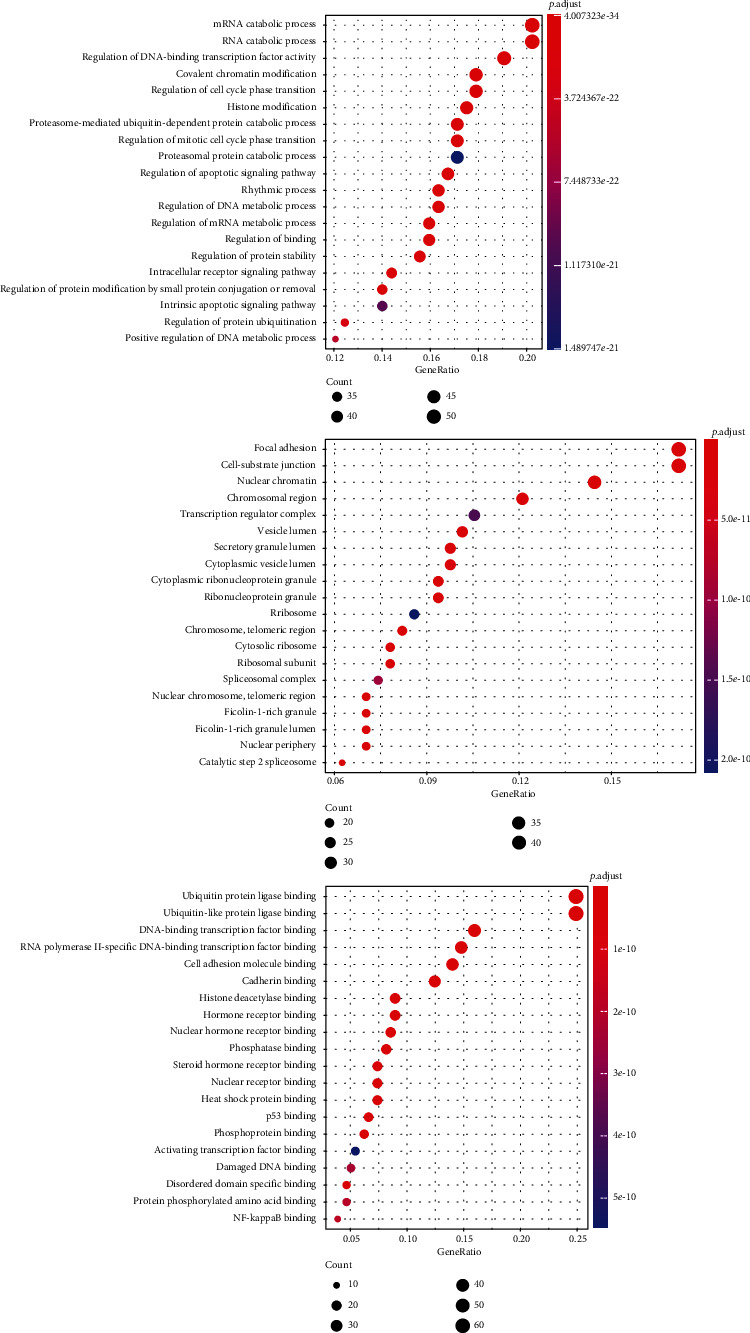
GO analysis of 285 candidate targets, BP, CC, and MF.

GO analysis of 285 candidate targets was analyzed based on biological process (BP), cellular component (CC), and molecular function (MF). 2,364 GO terms were finally enriched, 1,973 in biological process, 200 in cellular component, and 191 in molecular function. The top 20 terms are shown in Figure 8 (*P* value < 0.05). The results demonstrated many targets involved in multiple BPs associated with mRNA catabolic process, regulation of DNA-binding transcription factor activity, and regulation of cell cycle phase transition, which confirmed the correlation with the gene transcriptional activity in SLE-ONFH. The CC results suggested that most targets are localized to the focal adhesion, cell-substrate junction, and nuclear chromatin. The MF results showed that many targets are associated with ubiquitin protein ligase binding and DNA-binding transcription factor binding.

#### 3.3.2. KEGG Pathway Analysis

**Figure 9:**
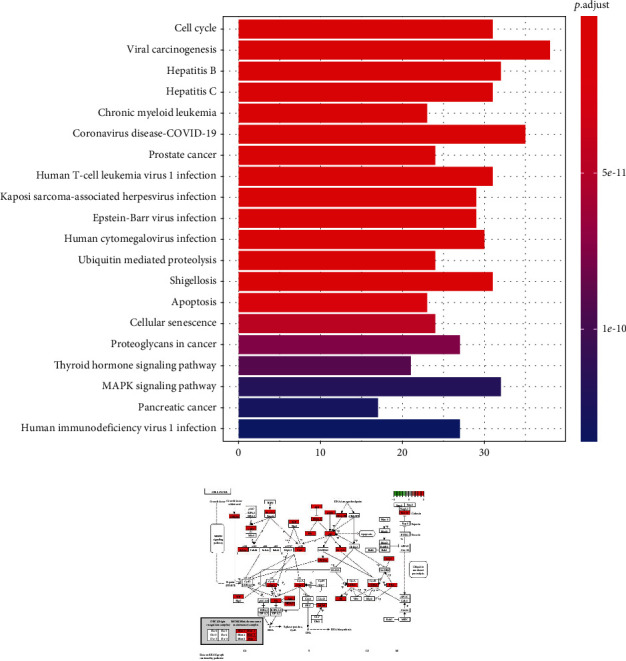
(a) KEGG analysis of 285 candidate targets; (b) cell cycle signaling pathway. The central gene p53 plays a pivotal role in the pathway.

The pathways that are significantly influenced by curcumin in the process of treating SLE-ONFH were identified by KEGG pathway analysis; the analysis method is similar to the GO analysis by running the corresponding script in R software. The top 20 significant pathways (*P* value < 0.05) are identified and shown in Figure 9(a), including cell cycle, viral carcinogenesis, hepatitis B, hepatitis C, chronic myeloid leukemia, and human T-cell leukemia virus 1 infection pathways; like the GO analysis above, they were closely related to cell proliferation, differentiation, senescence, and apoptosis. The most significant signaling pathway is the cell cycle pathway, in which the central genes such as TP53 play the pivotal role as shown in Figure 9(b). The data of analysis are shown in Table 3.

### 3.4. Gene-Pathway Network Analysis

**Figure 10:**
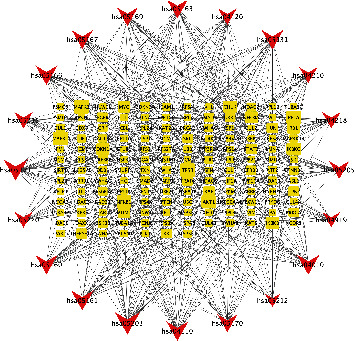
Gene-pathway network of SLE-ONFH.

The gene-pathway network was constructed based on the significantly enriched pathways and genes that regulated these pathways, which are presented in Figure 10. Topological analysis of 20 pathways and 285 candidate genes was carried out with BC. The squares represented target genes, and the V shapes represented pathways in the network. The network diagram suggested that TP53 has the most maximum BC and was the core target gene. Other several genes also had larger BC, such as HSP90AA1, GAPDH, MYC, and RPS27A. They might be the key target genes for the regulatory pathways of curcumin against SLE-ONFH.

### 3.5. Molecular Docking

**Figure 11:**
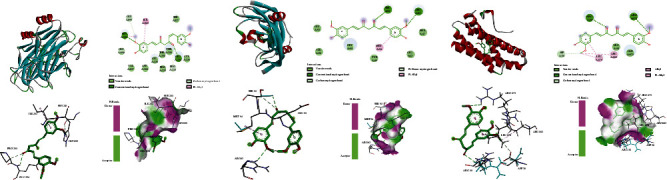
(a) Molecular docking between curcumin and TP53 (Affinity, -6.5). (b) Molecular docking between curcumin and VEGFA (Affinity, -5). (c) Molecular docking between curcumin and IL6 (Affinity, -5.9).

Molecular docking was applied to validate the binding action mode of the top three genes (TP53, VEGFA, and IL6), according to the network analysis. The results revealed that they can interact with curcumin; the combination of curcumin with them is shown in Figure 11. For curcumin and TP53, a ring forms a hydrophobic interaction with ILe232, a hydrogen bond and a hydrophobic interaction with HIS233, and a hydrogen bond with GLY199; the carbonyl group between the a and b rings forms a hydrogen bond with GLU224 and PRO223 (Figure 11(a)). For curcumin and VEGFA, a ring forms a hydrogen bond with GLU93, and the carbonyl group between the a and b rings forms a hydrogen bond with SER95; the b ring forms a hydrophobic interaction with MET94 and a hydrogen bond with ARG82 (Figure 11(b)). For curcumin and IL6, a ring forms a hydrophobic interaction with LEU178 and ARG182 and forms a hydrogen bond with ASP26, the carbonyl group between the a and b rings forms a hydrogen bond with ARG30, and the b ring forms a hydrogen bond with ARG179 (Figure 11(c)). The Affinity (kcal/mol) of curcumin and TP53, VEGFA, and IL6 was -6.5, -5, and -5.9, respectively, which means they can interact well indeed.

## 4. Discussion

### 4.1. Role of Network Pharmacology and Molecular Docking Strategy in the Research of Autoimmune Diseases

SLE is an autoimmune disease with multiple organ involvement and multiple antibodies. It is an urgent problem in the field of medicine. High disease activity of SLE is closely related to osteonecrosis especially ONFH, which has become the main cause of disability in SLE patients. In the whole process of disease development, immunity, inflammation, apoptosis, and angiogenesis are important factors [50–54]. Because of the complexity of its treatment, it is more worthwhile to find the appropriate drugs and explore the mechanism(s) than the final operation. Traditional Chinese medicine (TCM) has the characteristics of multitargets and multipathway effect, which may be more effective for complex diseases. As a drug research strategy, network pharmacology has been widely applied in drug research, which gives us a better understanding of the role of drug and their compounds [55]. In addition, molecular docking can help reveal the relationship between the drug and the target. Hence, in the present study, combined with topology analysis and molecular docking strategy, we used network pharmacology to investigate the potential targets involved in curcumin against SLE-ONFH by constructing and analyzing target networks, performing enrichment analysis with targets, and revealing the potential mechanism of curcumin against SLE-ONFH.

### 4.2. Analysis of Central Genes and Significant Pathways of Curcumin against SLE-ONFH

Curcumin has been found to be useful in the treatment of SLE and ONFH, but the central genes and significant pathways of curcumin against SLE-ONFH are not clear. Through the target interaction network, we found 36 potential targets as described in Results; there were 16 targets (TP53, VEGFA, IL6, TNF, EGFR, CASP3, ESR1, MMP9, CCND1, TGFB1, IL1B, CXCL8, FGF2, SRC, MMP2, and SERPINE1) with connectivity greater than the median degree. The TP53 gene is at the core of the whole network, which means that it is a central target. p53 protein is a tumor suppressor that inhibits the growth of aberrant cells; functional p53 is believed to sense DNA damage and, subsequently, to induce DNA repair, growth arrest, or apoptosis of the aberrant cell [56, 57]. On the one hand, as a cell cycle protein, p53 inhibits the proliferation of bone marrow-derived mesenchymal stem cells (BM-MSCs) of SLE patients. Previous studies have shown that p53 protein is highly expressed in SLE patients compared with the control group, and the activation of the p53 mediated signaling pathway was closely associated with the senescence of BM-MSCs from SLE patients [58]. On the other hand, it is wildly known that the function of BM-MSCs is closely related to the pathology of ONFH [59]. One study shows that p53 and Parkin that coregulate mitophagy in BM-MSCs could promote the repair of early steroid-induced ONFH [60]. Hence, it is at least indicated that p53 and its associated cell cycle signaling pathways are associated with BM-MSCs in SLE-ONFH patients. What is more, as an effective drug for the treatment of SLE and ONFH, curcumin could improve the function of BM-MSCs at the same time and is more likely to play a role by targeting p53 and related cell cycle signal pathway proteins. Of course, it also needs to be further determined by in vivo or in vitro experiments. In the network above, the second central gene is VEGFA. VEGFA is a highly specific vascular endothelial growth factor, which is involved in the occurrence and development of many angiogenesis-dependent diseases. A study found that the serum VEGF level in the active SLE group was significantly higher than that in the control and inactive SLE groups [51]. Moreover, it is reported that SNP rs2010963 of VEGFA is significantly associated with the risk of ONFH [61]. It is worth mentioning that VEGFA is also significantly associated with other autoimmune diseases such as rheumatoid arthritis [62]. IL6 and TNF are important cytokines involved in the immunopathological mechanism of SLE [63, 64]; they mediate the cellular inflammatory process. EGFR, epidermal growth factor receptor, which is Bsr I polymorphism is associated with SLE [65], and it also influences bone formation [66]. The process of ONFH is manifested by the apoptosis of bone cells, CASP3, as an extremely important molecule for apoptosis, which participated in the apoptosis process of bone-related cells [67]. MMP2 and MMP9 are members of the matrix metalloproteinase family. Studies have found that they are susceptibility genes for ONFH. MMP2 is known to be increased in osteoarthritic cartilage while MMP-9 regulates apoptosis of hypertrophic chondrocytes and is a key regulator of growth plate angiogenesis, thus rendering MMP-9 essential for normal bone development and remodeling [68]. ESR-1 and IL1B are crucial metabolism and inflammatory molecules; they are involved in the process of osteoblast-osteoclast metabolism and inflammation [69, 70]. Previous studies have also shown that they are associated with susceptibility to SLE [71–73]. CCND1, a cyclin indispensable for cell proliferation, participates in cell cycle regulation [74]. TGFB1, transforming growth factor-*β*, is associated with angiogenesis and vascularization and facilitates inflammation and joint damage [75]. CXCL8 is a member of the CXC family of chemokines, which is expressed in the kidneys of SLE patients. A meta-analysis showed that SLE patients have higher circulating IL-8 levels than normal controls and IL-8 levels in SLE patients are influenced by age, region, and disease duration [76]. FGF2 is a possible marker of kidney damage in SLE. SRC, the first identified viral oncogene, encoding tyrosine-protein kinase, has been reported to cause autoinflammatory bone disease [77]. SERPINE1 is also known as the plasminogen activator inhibitor-1; previous studies have demonstrated that decreased fibrinolytic activity due to elevated plasminogen activator inhibitor-1 (PAI-1) levels correlates with ONFH pathogenesis [78].

### 4.3. Research Value and Bright Prospects for Revealing the Targets and Mechanism of Curcumin against SLE-ONFH

At present, the treatment of SLE depends on the organ involved in the disease. Patients with mild symptoms can use low-dose corticosteroids; however, moderate and severe SLE may require higher doses of corticosteroids or other immunosuppressive agents. The adverse effects and limitations of hormones and immunosuppressive agents make TCM become a candidate therapeutic drug because of their unique advantages. The characteristic of SLE is that the complement system is activated; curcumin could inhibit the complement cascade. Studies have found that curcumin inhibits the increase of matrix protein, glial fibrillary acidic protein, and vimentin in the hippocampus of lupus mice [79]. As mentioned above, we found that curcumin may be able to treat SLE-ONFH by regulating TP53, involving the cell cycle pathway. As we all know, p53 protein plays a very important role in controlling the cell cycle, apoptosis, and DNA repair. There are abnormalities in BM-MSCs in SLE patients. Curcumin could have a positive effect on the treatment of SLE-ONFH by influencing the fate of BM-MSCs in SLE-ONFH patients. This provides an idea for the study of small molecular compounds specifically targeting p53 protein in SLE-ONFH disease. We initially confirmed this through molecular docking strategy. Combined with literature review, we found that curcumin could stabilize p53 by interaction with NAD(P)H: quinone oxidoreductase 1 in tumor-derived cell lines. Above all, according to our analysis, curcumin regulates p53 and influences the cell cycle. It may also regulate VEGFA, IL6, ERK1/2 (MAPK1/MAPK3), AKT1, MYC, CDKN1A, NF-KB, and other genes to effectively against SLE-ONFH because they have an important position in the gene-pathway network; this requires further research in the future, and we will continue to explore their roles in the process in SLE-ONFH.

## Figures and Tables

**Table 1:** Topological analysis of curcumin target network.

Name	ASPL	BC	CC	Clustering coefficient	Degree
TP53	2.015625	0.11128227	0.49612403	0.20244898	50
SRC	2.05729167	0.1213118	0.48607595	0.18454106	46
STAT3	2	0.08600855	0.5	0.23809524	43
AKT1	2.078125	0.08456637	0.48120301	0.20557491	42
MAPK3	2.06770833	0.0426935	0.4836272	0.26666667	40
MAPK1	2.05208333	0.07932813	0.48730964	0.25	40
EP300	2.125	0.09219908	0.47058824	0.20769231	40
JUN	2.0625	0.05135675	0.48484848	0.28609987	39
RELA	2.16666667	0.03133128	0.46153846	0.3125	32
VEGFA	2.11979167	0.04222026	0.47174447	0.27513228	28
TNF	2.25	0.03547444	0.44444444	0.29365079	28
FOS	2.1875	0.02564237	0.45714286	0.33903134	27
HSP90AA1	2.13541667	0.04893392	0.46829268	0.2991453	27
MAPK14	2.18229167	0.02577391	0.45823389	0.31054131	27
MYC	2.27604167	0.01608063	0.43935927	0.38666667	25
RB1	2.29166667	0.02207007	0.43636364	0.4	25
CCND1	2.27604167	0.01473079	0.43935927	0.40333333	25
EGFR	2.22916667	0.03631959	0.44859813	0.30434783	24
CDKN1A	2.34375	0.01530251	0.42666667	0.46014493	24
CDK1	2.53645833	0.01897448	0.39425051	0.43478261	23
ESR1	2.19270833	0.05987742	0.45605701	0.37944664	23
JAK2	2.40625	0.01035399	0.41558442	0.29437229	22
IL6	2.40625	0.0188487	0.41558442	0.37662338	22
CASP3	2.44791667	0.03709723	0.40851064	0.1991342	22
CXCL8	2.36458333	0.03987748	0.42290749	0.33766234	22
CASP8	2.40625	0.02257322	0.41558442	0.28095238	21
MMP9	2.48958333	0.0374213	0.40167364	0.29473684	20
CCNA2	2.77083333	0.00477339	0.36090226	0.47894737	20
CDK2	2.57291667	0.00859466	0.38866397	0.52105263	20
PTK2	2.328125	0.0222	0.4295302	0.26315789	19

**Table 2:** The potential targets of curcumin-SLE-ONFH (36 intersection genes).

Gene names	Annotation	Degree
TP53	Tumor protein p53	35
VEGFA	Vascular endothelial growth factor A	34
IL6	Interleukin 6	33
TNF	Tumor necrosis factor	32
EGFR	Epidermal growth factor receptor	31
CASP3	Caspase 3	31
ESR1	Estrogen receptor 1	29
MMP9	Matrix metallopeptidase 9	29
CCND1	Cyclin D1	28
TGFB1	Transforming growth factor beta 1	27
IL1B	Interleukin 1 beta	27
CXCL8	C-X-C motif chemokine ligand 8	27
FGF2	Fibroblast growth factor 2	26
SRC	SRC protooncogene, nonreceptor tyrosine kinase	26
MMP2	Matrix metallopeptidase 2	26
SERPINE1	Serpin family E member 1	26
KDR	Kinase insert domain receptor	25
TLR4	Toll like receptor 4	25
PPARG	Peroxisome proliferator-activated receptor gamma	25
PECAM1	Platelet and endothelial cell adhesion molecule 1	25
TIMP1	TIMP metallopeptidase inhibitor 1	24
MMP3	Matrix metallopeptidase 3	24
HIF1A	Hypoxia inducible factor 1 subunit alpha	24
NGF	Histone acetyltransferase GCN5	21
EP300	E1A binding protein p300	19
COL1A1	Collagen type I alpha 1 chain	18
MPO	Myeloperoxidase	17
SOD1	Superoxide dismutase 1	15
NOS2	Nitric oxide synthase 2	15
TGFBR1	Transforming growth factor beta receptor 1	15
ABCB1	ATP binding cassette subfamily B member 1	14
CYP19A1	Cytochrome P450 family 19 subfamily A member 1	11
CYP3A4	Cytochrome P450 family 3 subfamily A member 4	8
TNFRSF10A	TNF receptor superfamily member 10a	8
BAX	BCL2 associated X, apoptosis regulator	6
PCNA	Proliferating cell nuclear antigen	6

**Table 3:** The enrichment pathways corresponding to genes after topology.

Term	Description	*P* value
hsa04110	Cell cycle	4.78*E* − 22
hsa05203	Viral carcinogenesis	5.78*E* − 22
hsa05161	Hepatitis B	2.19*E* − 19
hsa05160	Hepatitis C	8.51*E* − 19
hsa05220	Chronic myeloid leukemia	1.22*E* − 18
hsa05171	Coronavirus disease—COVID-19	3.35*E* − 17
hsa05215	Prostate cancer	3.78*E* − 17
hsa05166	Human T-cell leukemia virus 1 infection	1.58*E* − 14
hsa05167	Kaposi sarcoma-associated herpesvirus infection	2.46*E* − 14
hsa05169	Epstein-Barr virus infection	8.30*E* − 14
hsa05163	Human cytomegalovirus infection	2.22*E* − 13
hsa04120	Ubiquitin mediated proteolysis	2.53*E* − 13
hsa05131	Shigellosis	4.02*E* − 13
hsa04210	Apoptosis	1.11*E* − 12
hsa04218	Cellular senescence	2.92*E* − 12
hsa05205	Proteoglycans in cancer	5.24*E* − 12
hsa04919	Thyroid hormone signaling pathway	6.69*E* − 12
hsa04010	MAPK signaling pathway	9.23*E* − 12
hsa05212	Pancreatic cancer	1.05*E* − 11
hsa05170	Human immunodeficiency virus 1 infection	1.17*E* − 11

## Data Availability

The data that support the findings of this study are available from the corresponding authors upon request.
